# Oral β-hydroxybutyrate increases ketonemia, decreases visceral adipocyte volume and improves serum lipid profile in Wistar rats

**DOI:** 10.1186/s12986-017-0184-4

**Published:** 2017-04-24

**Authors:** Rennan de Oliveira Caminhotto, Ayumi Cristina Medeiros Komino, Flaviane de Fatima Silva, Sandra Andreotti, Rogério Antônio Laurato Sertié, Gabriela Boltes Reis, Fabio Bessa Lima

**Affiliations:** Department of Physiology and Biophysics, Institute of Biomedical Sciences, University of Sao Paulo, Av. Prof. Lineu Prestes 1524 - Ed. Biomédicas I sala 131, Butantã, 05508-900 São Paulo Brazil

**Keywords:** β-hydroxybutyrate salts, Ketosis, HDL-cholesterol, Visceral fat, Experimental models

## Abstract

**Background:**

Ketosis can be induced in humans and in animals by fasting or dietary interventions, such as ketogenic diets. However, the increasing interest on the ketogenic state has motivated the development of alternative approaches to rapidly increase ketonemia using less drastic interventions. Here, it was tested whether oral intake of a β-hydroxybutyrate (βHB) mineral salt mixture could increase ketonemia in Wistar rats without any other dietary changes, thereby being a useful model to study ketones effects alone on metabolism.

**Methods:**

βHB salts were orally administered to provoke elevation in the ketonemia. Effects of this intervention were tested acutely (by gavage) and chronically (4 weeks in drinking water). Acutely, a concomitant glucose overload was used to suppress endogenous ketogenesis and verify whether βHB salts were really absorbed or not. Long-term administration allowed to weekly evaluate the impact on ketonemia, blood glucose and, after 4 weeks, on body weight, visceral fat mass, lipid blood profile, serum lipolysis products and adiponectinemia.

**Results:**

βHB salts increased ketonemia in acute and long-term administrations, improved blood lipid profile by raising HDL-cholesterol concentration and decreasing LDL/HDL ratio, while reduced visceral adipocyte volume. Mean ketonemia correlated positively with HDLc and negatively with adipocyte volume and serum lipolysis products.

**Conclusions:**

Oral βHB can rapidly increase ketonemia and, therefore, be used as an acute and long-term animal model of ketosis. Long-term treatment points to important beneficial effects of ketone bodies in serum lipid concentrations and visceral fat mass. These results may help to explain the metabolic adaptations following ketogenic diets, such as a better body fat control and a serum lipid profile improvement.

## Background

It has been demonstrated that ketogenic diets have beneficial effects to overweight [1, 2], especially in insulin resistance subjects [3, 4]. Body weight and fat loss, reduced glycemia, improvement of blood lipid profile by increasing HDL-cholesterol and decreasing triglycerides are among the most reproduced metabolic benefits of ketogenic diets [5]. Also, there is evidence that blood adiponectin, an adipokine that enhances insulin sensitivity and exerts an anti-inflammatory effects [6], can be improved by ketogenic diets in children [7].

However, the dietary carbohydrate restriction associated with ketogenic diets by itself and the parallel increase in protein/lipid consumption, as well as the state of nutritional ketosis, are all potential mechanisms to explain the better weight control, blood lipid profile and glycemic improvement seen in protocols which use ketogenic diets. In this sense, few works have dealed with more direct and strict effects of ketones in evoking the above clinical picture.

Exogenous administration of ketones has appeared as an interesting tool to study and reproduce isolated ketone effects. Recently, Kesl et al. [8] have tested several natural and synthetic ketone supplements in Sprague–Dawley rats, where ketosis was tried to be experimentally induced using 1,3-butanediol, medium chain triglyceride oil (MCT), a ketone ester or β-hydroxybutyrate (βHB) mineral salts. Among the tested supplements, only βHB mineral salts were not able to induce elevation in ketonemia in Sprague–Dawley rats. Nevertheless, βHB mineral salts supplementation has produced a significant hypoglycemic effect when in combination with MCT oil while alone decreased the body weight gain after 4 weeks of treatment, all aspects that are influenced by ketogenic diets.

Oral βHB salts intake has already been reported in some previous studies: It was already effective and safely used for the treatment of 1 year-old child with hypoketotic hypoglycemia caused by a fatty acid oxidation disorder [9]; to increase blood and cerebrospinal fluid ketone concentrations in two infant patients with persistent hyperinsulinemic hypoglycemia [10]; in the treatment of multiple acyl-CoA dehydrogenase deficiency (MADD) in three infants (a child with leukodystrophy and two infants with cardiomyopathy) [11]; and more recently, in a case of MADD-related leukodystrophy during 3 years of treatment [12]. All of that circumstances evidence the capacity of absorption and utilization of oral βHB salts, at least in humans.

Therefore, considering the previous studies cited above [9–12], we decided to retest the hypothesis that βHB mineral salts could increase ketonemia in a different animal model (Wistar rats) and assess whether supplemented oral βHB mineral salts could be a model of chronic ketosis. Also, we intended to describe whether βHB mineral salts could influence others parameters improved by ketogenic diets, as body fat and adiponectinemia, as well blood glucose and lipid profile.

## Methods

### Acute administration of βHB mineral salts

Animal procedures were performed in accordance with the Ethics Committee on Animal Use of Institute of Biomedical Sciences of the University of São Paulo (18/2016). Ten Wistar rats (8–9 weeks old), after 6 h of fasting, in Zeitgeber time 0 (experimental dawn [lights on]), were divided into two weight-matched groups: (1) βHB salt group, which received a single dose experiment by gavage a mixture of βHB sodium and potassium salt form, in the concentrations of 300 mg/100 g b.w. of βHB; and (2) 41 mg/100 g b.w. of sodium and potassium. βHB sodium/potassium supplement were purchase from KetoSports (KetoForce by Prototype Nutrition, Savind Inc - IL, USA) and consist in a solution of approximately 50% of βHB sodium and potassium salt. Control group received a salt mixture (NaCl/KCl) in equivalent concentrations of sodium/potassium and volume founded in βHB mineral salts supplement. In addition, the two groups received a concomitant glucose overload (75 mg/100 g b.w.) with a 50% glucose solution, with the intent to suppress endogenous βHB formation during the test, in order to better demonstrate whether ketosis were really being induced by exogenous βHB mineral salts. Tail blood was collected by small cut in tail tip. Ketonemia and glycemia were measured at baseline (time 0) immediately prior to gavage and at the following times (15′, 30′, 60′, 120′). Ketonemia was measured by Freestyle Optium XCEED (Abbott Diabetes Care, Alameda, CA, USA) and Freestyle Optium β-Ketone strips. Glycemia was measuring using a glucometer (One Touch Ultra LifeScan, Milpitas, CA, USA).

### Oral chronic treatment with βHB mineral salts

Wistar rats (10 weeks old) were weight paired and assigned to control (*n* = 9) or βHB salts (*n* = 10) group, which received a βHB mineral salts solution in drinking water (final concentration of 4.2%) for 4 weeks. This concentration was chosen considering a final isotonic concentration of sodium and potassium sum (0.45% of each one). Drinking solutions were fully available throughout the experimental period. Ketonemia and glycemia were measured weekly as described above, between Zeitgeber hours (ZT) 14–15 (experimental night) and 0–1 (experimental morning). Body weight, food and caloric intake (including βHB mineral salts calories) were also measured.

### Euthanasia

Fasted rats (12 h) were anesthetized with thiopental sodium (50 μg/g b.w.) prior to the euthanasia performed by decapitation at ZT 13–15. Water and βHB salts solution remained available during the overnight fasting. Blood serum was collected for biochemical analyses. The visceral mesenteric fat pad was collected and weighed.

### Determination of adipocyte volume

Adipocytes from visceral mesenteric fat pad were isolated as previously described by Rodbell [13], and fixed in 4% formaldehyde. Fixed fat cells were then photographed (×100) using a microscope camera and its diameter were determined using the software Motic-Images Plus 2.0®. A mean of 100 cells was used. Volume was calculated as previously described by Di Girolamo et al. [14].

### Blood lipid and lipolysis products measurement

Enzymatic colorimetric assays of Triacylglycerol (TAG–Liquiform-Labtest 87), total cholesterol (TC–Liquiform -Labtest 76) and high-density lipoprotein fraction (HDL- Liquiform-Labtest 13) were used to determinate blood lipids concentrations. Low-density lipoprotein fraction was calculated using Friedewald equation (LDL = Total Cholesterol - HDL – [Triglycerides/5]). Glycerol and FFA was also measured using an enzymatic-colorimetric methods (Free Glycerol Determination Kit - Sigma-Aldrich; Wako HR series NEFA-HR-2).

### Adiponectin determination in blood and adipose tissue

Adiponectinemia and adipose tissue adiponectin protein concentration were meansured by an adiponectin rat ELISA kit (CUSABIO CBS-E07271r).

### Statistics analysis

All data are presented as the mean ± standard error of mean (SEM). Data analysis was performed using GraphPad PRISM® version 5. Results were considered significant when *p* < 0.05. Student’s t test was used to compare control and treated groups. Two-Way ANOVA was used to compare curve data. Correlations were done by a linear regression of correlations by GraphPad PRISM®. Bonferroni post hoc test was used in all ANOVA analyses.

## Results

### Oral βHB impact on ketonemia

**Fig. 1:**
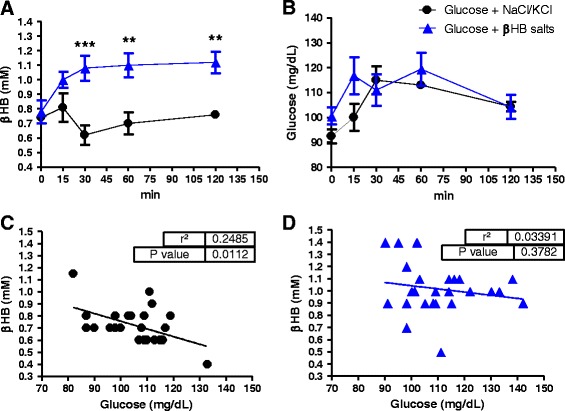
Oral β-hydroxybutyrate increases ketonemia: **a** βHB salts significantly increased ketonemia after 30 min and sustained it for at least 2 h; **b** βHB salts did not change glycemia during glucose overload (75 mg/100 g b.w.); **c**, **d** βHB salts disrupts negative correlation between glycemia and ketosis after glucose overload (*p* = 0.378). *n* = 5 each group in all data. Two-Way ANOVA with Bonferroni post hoc test. **p < 0.01; ***p < 0.001. Data represent as mean (SEM)

Oral administration (by gavage) of βHB salts increased ketonemia (Fig. 1a) compared to control during all the time tested (2 h), even during concomitant administration of glucose (Fig. 1b) intended to suppress endogenous ketogenesis, showing that elevation of blood βHB was caused by βHB exogenous supplementation. Blood glucose, as expected, correlated negatively with βHB levels in Control group (Fig. 1c), showing that during the oral test the rise in glycemia was accompanied by a reduction in ketosis. On the other hand, in βHB salt supplemented rats, there was a disruption of this inverse relationship because an elevation in ketonemia occurred concomitantly with the increase in glycemia (Fig. 1d).

### Chronic βHB supplementation kept ketonemia increased throughout the day

**Fig. 2:**
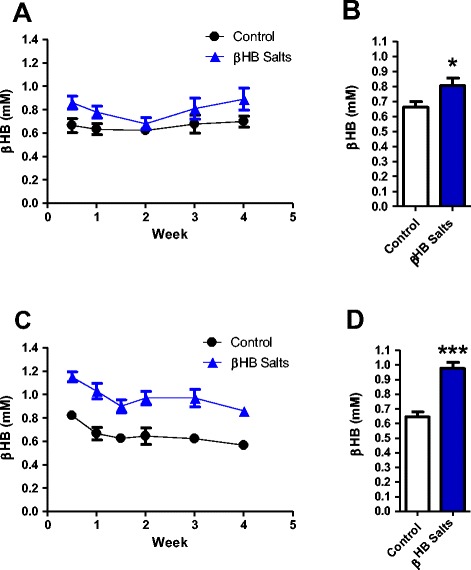
Continuously β-hydroxybutyrate intake increases ketonemia along the day: **a**, **b** Rats continuously drinking βHB solution had a mean increase (22%) in ketonemia during experimental evening (ZT 14–16), while **c**, **d** a 51% increase was seen during experimental morning (ZT 0–1). *n* = 9-10 in all data. Student’s t test. *p < 0.05; ***p < 0.001. Data represent as mean (SEM)

Continues ad libitum administration of 4.2% βHB solution increased ketonemia during the two different daily periods analyzed. Ketone bodies were weekly determined between ZT 14–16 (lights off) and 0–1 (lights on). The average determination of blood ketone bodies along the 4 weeks was about 22% higher in βHB supplemented vs. control rats in ZT 14–16 (Fig. 2b), while the ketonemia values obtained at ZT 0–1 were 51% more elevated in average during the 4-week period in the supplemented animals (Fig. 2d). Daily intake of βHB averaged was ~600 mg/100 g body weight.

### βHB mineral salts did not change body weight and caloric intake

Continues ad libitum administration of water with 4.2% of βHB salts did not significantly change body weight, food consumption and caloric intake after 4 weeks of treatment (Table 1.).

**Table 1:** Comparations of body weigth, food and caloric intake

	Control	βHB Salts	*P* value
Initial body weight (g)	308.6 (7.6)	304.0 (6.4)	0.6466
Final body weight (g)	370.1 (9.7)	358.2 (8.9)	0.3825
Food Intake (g/day)	22.9 (1.0)	20.8 (0.6)	0.0836
Caloric Intake (Kcal/day)	94.0 (4.2)	96.6 (2.5)	0.5883

### βHB mineral salts improved lipid profile

**Fig. 3:**
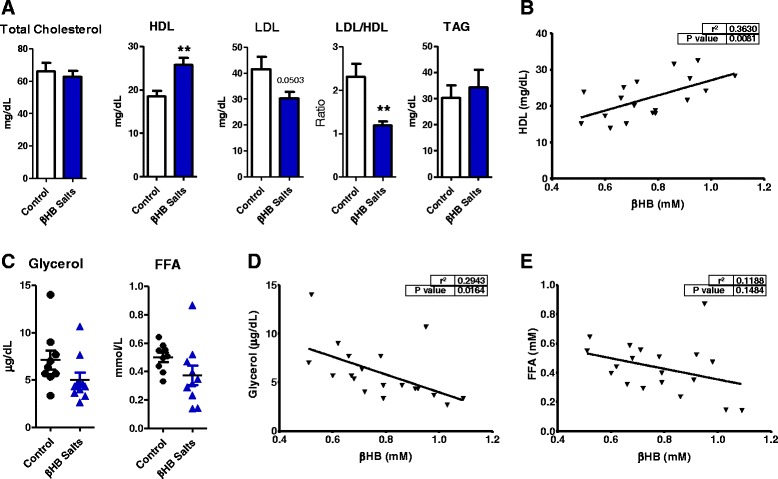
β-hydroxybutyrate improved lipid profile and appears to influence serum lipolysis products: **a** Rats which drank βHB solution had improvements in LDL (−35%) and HDL (+39%) cholesterol, **b** with a positive correlation between HDL cholesterol and mean of ketonemia (*p* > 0001). Also, **c** despite lack of significantly changes, βHB salts treatment appears to influence serum lipolytic products, glycerol and FFA, **d** with a negative correlation between glycerol and mean ketonemia values (*p* < 0.05). **e**﻿ Although there is no significance in FFA data. *n* = 9-10 in all data except in HDL cholesterol (*n* = 8-10). Student’s t test. **p < 0.01. Data represent as mean (SEM)

Chronic βHB supplementation improved lipid profile after 4 weeks of treatment. HDL cholesterol increased 39% while LDL decreased 35% leading to a 49% reduction of the LDL/HDL ratio (Fig. 3a). Also, a positive correlation between blood HDL cholesterol and ketone bodies levels was found (Fig. 3b).

### βHB mineral salts diminished visceral adipocytes volume

**Fig. 4:**
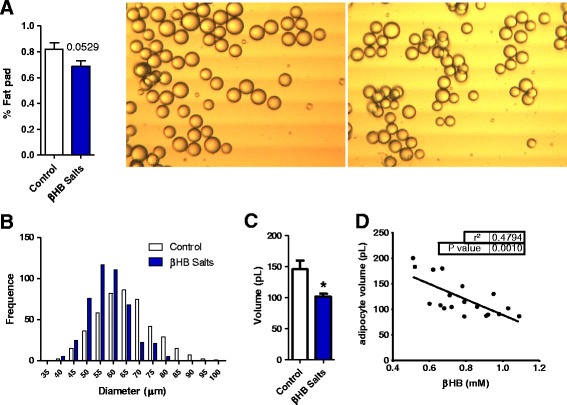
β-hydroxybutyrate decreases visceral adipocyte volume: **a** Rats had a decreased percentage of visceral fat (−16%) and **b**, **c** and image [control in *left*, treatment on *right*]) a small adipocyte volume (−30%) after 4 weeks of treatment. **d** A negative correlation between cell volume and mean of ketonemia were found (*p* > 0,01). *n* = 9-10. Student’s t test *p < 0.05. Data represent as mean (SEM)

The percentage of visceral fat mass of βHB treated rats showed a tendency to a reduction (−16%) although the difference was not statistically significant (*p* = 0.0529) (Fig. 4a). However, their fat cells were 30% smaller (Fig. 4b, c and image). Lastly, a negative correlation between visceral adipocyte volume and average ketonemia were found.

### βHB mineral salts did not change blood glucose and adiponectin concentrations

**Fig. 5:**
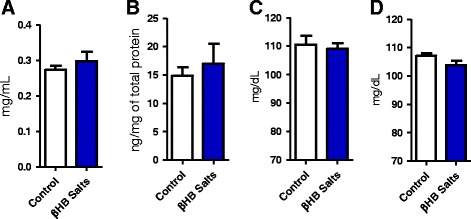
β-hydroxybutyrate and blood and tissue adiponectin: Adiponectin protein concentration did not have any changes in **a** blood or **b** visceral adipose tissue. *n* = 9. Also, blood glucose (4 weeks avarege - weekly measurements) has not changed in **c** experimental evening (ZT 14-16) or **d **experimental morning (ZT 0-1)﻿. n=9-10. Student’s t test. Data represent as mean (SEM)

In addition, βHB treatment did not influence adiponectinemia at all (Fig. 5a) nor the adiponectin protein content in visceral fat (Fig. 5b). The same happened with the blood glucose levels as a consequence of the treatment (Fig. 5c [ZT 14–15] and d [ZT 0–1]).

## Discussion

Different of the results of Kesl et al. [8] in Sprague Dawley rats, this study demonstrated that acute oral βHB mineral salts (3 g/kg) are really absorbed and reach the systemic circulation after gavage in Wistar rats and can rapidly increase ketonemia at levels similar to that found during nutritional ketosis [8]. Here, ketosis was maintained even during concomitant glucose intake, which is known to suppress ketogenesis due to the stimulation of endogenous insulin secretion [15], proving that exogenous ketones were the main and possibly the only source of ketone elevation above basal values in blood during the oral test. In the control group, on the contrary, levels of ketone bodies slightly went down during glucose ingestion only. Also, in chronic treatment, despite the ketonemia values have been lower than those achieved during the gavage test and the ketone concentration varied depending on the time of the day the ketone analysis was done (“lights on” values > “lights off”), the ketonemia values were always higher in the treated group. Therefore, this protocol is a useful long-term model to increase ketonemia, which is independent on carbohydrate intake or hepatic substrate metabolic fate (i.e. beta oxidation activation).

It is hypothesized that, once in bloodstream, exogenous βHB, like the endogenous ketones, is taken up by monocarboxylic acid transporters, metabolized to acetoacetate by D-β-hydroxybutyrate dehydrogenase and utilized as an energy source by extrahepatic tissues, such as heart, brain and muscle. However, some important differences between exogenous βHB administration (as βHB salts) and endogenous production (such as in fasting, ketogenic diets) are expected [16]. The strategy of oral supplementation of βHB salts provides the entry of this substrate without the need to synthesize it endogenously dispensing with the several steps of its production which include an increased glucagon/insulin ratio, lipolysis, transport of lipids to the liver and beta-oxidation. Thus, by using this protocol, it is possible to isolate ketone/ketosis effects and investigate whether the beneficial results frequently found during ketogenic diets are due to ketones directly or to other dietary variants, as higher protein/fat intake or carbohydrate restriction.

In addition, in our model, all signaling effects βHB can be expected, such as activation of GPR10A receptor [17]. βHB is an endogenous ligand of GPR10A receptor, which is also a receptor activated by niacin. Niacin is recognized as a pharmacological agent for the treatment of dyslipidemia, used to lower VLDL and LDL lipoproteins, while it raises HDL cholesterol [18]. Here, chronic βHB administration had effects in lipid profile, notably by raising HDL cholesterol and decreasing LDL/HDL ratio, suggesting that ketones may have similar effects of niacin on these parameters.

By sharing the same niacin receptor, this mechanism of βHB action may explain the improvement of blood lipids profile, since it has an inhibitory effect in lipolysis, through Gi protein activation and a subsequent decreasing release of serum lipolytic products, as free-fatty acids (FFA) and glycerol [17]. It is known that serum FFA directly affects other lipid transporters (VLDL/LDL lipoproteins) in blood, since it is a substrate for both triglyceride and cholesterol synthesis [18].

In our study, βHB chronic treatment appears to influence serum lipolytic products because ketonemia was negatively correlated with serum glycerol, a frequently used marker of lipolytic activity in vivo and in vitro. Evidently, our data reflect a transitory influence that might be more intense if considering other periods of the day (remembering that euthanasia and sample collection occurred at ZT 14–16, when ketonemia was lower compared to the levels found at ZT 0–1), and with a significant influence along the 4 weeks of treatment. Thus, studies are necessary to confirm that ketone supplementation runs with low lipolytic rates along the day. Anyway, a former study showed that a partial inhibition of adipocyte lipolytic rates, even without changes in basal glycerol or FFA serum concentrations, improved the animal’s metabolic conditions, such as the insulin tolerance in mice treated with hormone-sensitive lipase inhibitor [19]. It is important to figure out that the fluctuations of serum glycerol and FFA blood levels along the day are quite more complex than their release by adipocyte through lipolysis can explain. Glycerol concentrations also depend on its utilization by liver, where it is utilized as a gluconeogenic substrate. Also, above and beyond being utilized as an energy source, FFA can also be re-esterified back into triglycerides by adipocytes, in a triglyceride/fatty acid futile cycle. These points indicate that the in vivo estimation of lipolysis is not that simple, although an inhibitory effect by βHB can be hypothesized based on former in vitro and in vivo studies [20, 21].

However, in addition to what might be taking place in our protocol, a special attention is needed on the morphological changes in visceral fat considering the results from the lipid profile. The enlargement of the visceral fat cells denotes that an increased coming of FFA is occurring specifically through the mesenteric circulation toward the portal-hepatic system, and it is an important risk factor to ectopic liver fat deposition and hepatic metabolic dysfunctions [22, 23]. Therefore, our results indicate that both the smaller visceral fat cell volume found after chronic treatment and the decreased rates of lipolysis elicited by βHB treatment may have contributed to improvement of serum lipid profile. To reinforce this idea, enlarged adipocytes inversely correlate with insulin sensitivity [24] and positively with FFA release, particularly in visceral cells [25].

Regarding the HDL rise observed in βHB treated rats (Fig. 3a), it can be hypothesized that βHB can act like niacin by reducing the HDL catabolism and prolonging its half-life due to an inhibition of the hepatocyte lipoprotein uptake [26]. If the correctness of this supposition is confirmed, it could explain our data shown in Fig. 3. On the other hand, visceral fat deposition also has a well-known negative correlation with HDL cholesterol [27], and this is another way βHB treatment could be indirectly influencing the HDLc concentrations.

An additional biological effect of GPR10A activation is the increment of adiponectin secretion by adipocytes [17]. Like niacin, βHB is able to induce adiponectin secretion in primary adipocytes, although there are no data on whether this effect also happens in vivo when stimulated by ketones alone. On the other hand, ketogenic diet elevated adiponectinemia in children on a trial comparing caloric restriction and ketogenic diet [7]. Thus, these are enough evidences to lead us to hypothesize that βHB salts supplementation could increase adiponectin secretion. Furthermore, our verification of smaller fat cells in adipose tissue of βHB supplemented rats reinforce this supposition, since adiponectin synthesis and secretion is more intense in smaller fat cells. Fat depots containing a larger proportion of small adipocytes appear to have a higher ability to secrete adiponectin [28]. However, hyperadiponectinemia was not reported in our chronic protocol.

Blood glucose was another unaffected parameter in this study. In Kesl et al. [8], increased ketonemia was negatively correlated with blood glucose, suggesting a hypoglycemic effect. Indeed, Kesl et al. [8] obtained a higher ketonemia than we got here with the exogenous ketone supplements. Still, it is possible to note in Kesl’s work that ketone esters and specially MCT oil, which were the ketone supplements that most increased ketonemia, presented relevant suppression of blood glucose, perhaps demonstrating the need for a greater ketosis than that found in our experiment to achieve the same result. However, MCT oil could induce a lower glycemia by interfering with the dietary carbohydrate digestion and absorption rate or by a strong reduction in food consumption after gavage instead of its direct effect.

In our experiment, daily food consumption as well caloric intake did not change significantly (−9%, *p* = 0.08). Also different from βHB salts treated animals in Kesl et al. [8], our animals did not lose weight, although the percentage of visceral fat mass tended to be decreased (−16%, *p* = 0.0529). It should be noted that in our protocol rats received less βHB salts than in Kesl’s study (~6 g/kg vs. 5–10 g/kg, respectively). The different result between Kesl’s study and ours is possibly due to the different rat model in consideration (Sprague Dawley vs Wistar) as well the way of BHB administration (daily gavage vs drink water).

However, gathering all the results and comments plus the decreased fat cell volume found, interesting elements come out: ketogenic diets have already shown advantages in important clinical trials, at least in short/medium term, when carbohydrate ingestion is low enough to increases ketonemia [1, 2]; in mice, ketogenic diets decrease body weight and fat mass as well as a caloric restriction diet, even those with a higher caloric content per gram [29]; as Srivastava, et al. [30] reported, a shrinkage of body fat in mice fed a ketone ester/carbohydrate free diet and an increase resting energy expenditure and brown fat activity; rats fed on a diet enriched with 1,3-butanediol (a ketogenic alcohol) got a reduced body fat mass [31]. In our protocol, differently from the others above, since we did not make any change in animal diet and the caloric intake remained the same, an isolated ketotic effect was reached on visceral fat mass and tissue morphology. This data may help to explain the metabolic advantage (i.e. a better body weight/fat loss) found during ketogenic diets. Still, a dose-dependent effect of βHB salts might be possible, at least on ketonemia. For that, maybe other models of administration would be interesting, as βHB salts on food, as is being done by D’Agostino’s group [32]. Lastly, it is important to emphasize that although the amount of βHB salts supplemented here in rats was a useful tool for our experimental purposes, a translational approach to humans must be taken with care regarding the sodium and potassium ingestion and some concern must be considered. For example, in an average adult man (~70 kg), to keep the same proportions βHB salts, an ingestion of ~50 g of sodium and potassium would occur which is evidently well above the dose recommended by RDA.

## Conclusions

In conclusion, we demonstrated that βHB salts oral intake in drinking water is useful tool to obtain a long term model of elevated ketonemia in Wistar rats, without any dietary change, as carbohydrate restriction or high fat consumption. Also, our long term model of elevated ketonemia by administration of βHB salts point to important biological effects of ketone bodies in control of serum lipid concentrations, particularly HDL cholesterol and control of visceral fat mass content, specially by contracting the adipocyte size. These results help to explain some of metabolic adaptations that follow the introduction of ketogenic diets, such as the improvement of body control and a more effective reduction of fat mass and serum lipid profile amelioration. This and other ketone supplements may be helpful in the future to complement the treatment of obesity, metabolic syndrome and related diseases.

## Abbreviation

FFA: Free fatty acids
HDL: High density lipoprotein
LDL: Low density lipoprotein
MADD: Multiple acyl-CoA dehydrogenase deficiency
MCT: Medium chain triglyceride
ZT: Zeitgeber hours
βHB: Beta-hydroxybutyrate

## References

[1] Gardner CD, Kiazand A, Alhassan S, Kim S, Stafford RS, Balise RR (2007). Comparison of the Atkins, Zone, Ornish, and LEARN diets for change in weight and related risk factors among overweight premenopausal women: the A TO Z Weight Loss Study: a randomized trial. JAMA.

[2] Foster GD, Wyatt HR, Hill JO, McGuckin BG, Brill C, Mohammed BS (2003). A randomized trial of a low-carbohydrate diet for obesity. N Engl J Med.

[3] Hussain TA, Mathew TC, Dashti AA, Asfar S, Al-Zaid N, Dashti HM (2012). Effect of low-calorie versus low-carbohydrate ketogenic diet in type 2 diabetes. Nutrition.

[4] Cornier MA, Donahoo WT, Pereira R, Gurevich I, Westergren R, Enerback S (2005). Insulin sensitivity determines the effectiveness of dietary macronutrient composition on weight loss in obese women. Obes Res.

[5] Bueno NB, de Melo IS, de Oliveira SL, da Rocha Ataide T (2013). Very-low-carbohydrate ketogenic diet v. low-fat diet for long-term weight loss: a meta-analysis of randomized controlled trials. Br J Nutr.

[6] Proença AR, Sertié RA, Oliveira AC, Campaña AB, Caminhotto RO, Chimin P (2014). New concepts in white adipose tissue physiology. Braz J Med Biol Res.

[7] Partsalaki I, Karvela A, Spiliotis BE (2012). Metabolic impact of a ketogenic diet compared to a hypocaloric diet in obese children and adolescents. J Pediatr Endocrinol Metab.

[8] Kesl SL, Poff AM, Ward NP, Fiorelli TN, Ari C, Van Putten AJ, et al. Effects of exogenous ketone supplementation on blood ketone, glucose, triglyceride, and lipoprotein levels in Sprague–Dawley rats. Nutr Metab (Lond). 2016;13(9). 10.1186/s12986-016-0069-y PMC4743170 26855664

[9] Bonham JR, Tanner MS, Pollitt RJ, Manning NJ, Olpin SE, Downing M (1999). Oral sodium 3-hydroxybutyrate, a novel adjunct to treatment for multiple acyl CoA dehydrogenase deficiency. J Inherit Metab Dis.

[10] Plecko B, Stoeckler-Ipsiroglu S, Schober E, Harrer G, Mlynarik V, Gruber S (2002). Oral beta-hydroxybutyrate supplementation in two patients with hyperinsulinemic hypoglycemia: monitoring of beta-hydroxybutyrate levels in blood and cerebrospinal fluid, and in the brain by in vivo magnetic resonance spectroscopy. Pediatr Res.

[11] Van Hove JL, Grünewald S, Jaeken J, Demaerel P, Declercq PE, Bourdoux P (2003). D, L-3-hydroxybutyrate treatment of multiple acyl-CoA dehydrogenase deficiency (MADD). Lancet.

[12] Gautschi M, Weisstanner C, Slotboom J, Nava E, Zürcher T, Nuoffer JM (2015). Highly efficient ketone body treatment in multiple acyl-CoA dehydrogenase deficiency-related leukodystrophy. Pediatr Res.

[13] Rodbell M (1964). Metabolism of isolated fat cells. I. Effects of hormones on glucose metabolism and lipolysis. J Biol Chem.

[14] Di Girolamo M, Mendlinger S, Fertig JW (1971). A simple method to determine fat cell size and number in four mammalian species. Amer J Physiol.

[15] Soeters MR, Sauerwein HP, Faas L, Smeenge M, Duran M, Wanders RJ (2009). Effects of insulin on ketogenesis following fasting in lean and obese men. Obesity (Silver Spring).

[16] Cox PJ, Clarke K (2014). Acute nutritional ketosis: implications for exercise performance and metabolism. Extrem Physiol Med.

[17] Plaisance EP, Lukasova M, Offermanns S, Zhang Y, Cao G, Judd RL (2009). Niacin stimulates adiponectin secretion through the GPR109A receptor. Am J Physiol Endocrinol Metab.

[18] Carlson LA (2005). Nicotinic acid: the broad-spectrum lipid drug. A 50th anniversary review. J Intern Med.

[19] Girousse A, Tavernier G, Valle C, Moro C, Mejhert N, Dinel AL (2013). Partial inhibition of adipose tissue lipolysis improves glucose metabolism and insulin sensitivity without alteration of fat mass. PLoS Biol.

[20] Taggart AKP, Kero J, Gan Z, Cai TQ, Cheng K, Ippolito M (2005). (D)-β-Hydroxybutyrate inhibits Adipocytes Lipolysis via the Nicotinic Acid Receptor PUMA-G. J Biol Chem.

[21] Shaw JH, Wolfe RR (1984). Influence of beta-hydroxybutyrate infusion on glucose and free fatty acid metabolism in dogs. Am J Physiol.

[22] Nielsen S, Guo Z, Johnson CM, Hensrud DD, Jensen MD (2004). Splanchnic lipolysis in human obesity. J Clin Invest.

[23] Tchernof A, Després JP (2013). Pathophysiology of human visceral obesity: an update. Physiol Rev.

[24] Weyer C, Foley JE, Bogardus C, Tataranni PA, Pratley RE (2000). Enlarged subcutaneous abdominal adipocyte size, but not obesity itself, predicts type II diabetes independent of insulin resistance. Diabetologia.

[25] Gaidhu MP, Anthony NM, Patel P, Hawke TJ, Ceddia RB (2010). Dysregulation of lipolysis and lipid metabolism in visceral and subcutaneous adipocytes by high-fat diet: role of ATGL, HSL, and AMPK. Am J Physiol Cell Physiol.

[26] Kamanna VS, Kashyap ML (2008). Mechanism of action of niacin. Am J Cardiol.

[27] Rashid S, Genest J (2007). Effect of obesity on high-density lipoprotein metabolism. Obesity (Silver Spring).

[28] Meyer LK, Ciaraldi TP, Henry RR, Wittgrove AC, Phillips SA (2013). Adipose tissue depot and cell size dependency of adiponectin synthesis and secretion in human obesity. Adipocyte.

[29] Kennedy AR, Pissios P, Otu H, Roberson R, Xue B, Asakura K (2007). A high-fat, ketogenic diet induces a unique metabolic state in mice. Am J Physiol Endocrinol Metab.

[30] Srivastava S, Kashiwaya Y, King MT, Baxa U, Tam J, Niu G (2012). Mitochondrial biogenesis and increased uncoupling protein 1 in brown adipose tissue of mice fed a ketone ester diet. FASEB J.

[31] Clayton GD. and Clayton FE, editors. Patty’s Industrial Hygiene and Toxicology: Volume 2A, 2B, 2C: Toxicology. 3rd ed. New York: John Wiley Sons; 1981–1982. p. 3878.

[32] Ari C, Kovács Z, Juhasz G, Murdun C, Goldhagen CR, Koutnik AP (2016). Exogenous Ketone supplements reduce anxiety-related behavior in Sprague–Dawley and Wistar Albino Glaxo/Rijswijk Rats. Front Mol Neurosci.

